# Prevalence of Female Authors in Case Reports Published in the Medical Literature

**DOI:** 10.1001/jamanetworkopen.2019.5000

**Published:** 2019-05-31

**Authors:** David Hsiehchen, Antony Hsieh, Magdalena Espinoza

**Affiliations:** 1 Division of Hematology and Oncology, Department of Internal Medicine, University of Texas Southwestern Medical Center, Dallas; 2 Division of Gastroenterology, Department of Medicine, Perelman School of Medicine, University of Pennsylvania, Philadelphia; 3 Division of Gastroenterology, Department of Medicine, Banner University Medical Center–Phoenix, University of Arizona, Phoenix

## Abstract

**Importance:**

Underrepresentation of female authors in research publications is prevalent, but it is unclear whether this is attributable to sex disparities in research conduct or authorship practices. Case reports are a poorly understood component of the biomedical corpus, and the production of anecdotal observations is not confounded by factors associated with disparities in female representation in research publications. Whether female authorship disparities exist in nonresearch publications of clinical information is unknown.

**Objectives:**

To examine the authorship of case reports and elucidate factors associated with sex disparity.

**Design and Setting:**

Cross-sectional study of all case reports published by US authors in 2014 and 2015 indexed in PubMed performed from July 2015 to July 2018.

**Main Outcomes and Measures:**

The primary outcome measure was the proportion of female first authors. The secondary outcome measures were the proportion of female last authors and female authorship representation among different clinical specialties.

**Results:**

Bibliometric data was abstracted from 20 427 case reports published across 2538 journals. A total of 7252 (36%) and 4825 (25%) case reports had a female first and last author, respectively. In comparison, 44% and 34% of US trainees and physicians, respectively, were female in 2015. Among adult case reports, female authorship was more prevalent in academic environments compared with community settings (34.0% vs 28.2% for female first authors and 23.4% vs 19.7% for female last authors). Across states, the proportions of female first authors and last authors were universally less than the proportions of female trainees and active female physicians, respectively. Female first authorship was associated with larger author teams (odds ratio [OR], 1.02; 95% CI, 1.01-1.03), an academic affiliation (OR, 1.16; 95% CI, 1.06-1.27), and a female last author (OR, 1.58; 95% CI, 1.47-1.70). Relative to general internal medicine, specialties dominated by male clinicians were less frequently associated with female first authors. Several exceptions displaying a relatively equivalent tendency for male and female first authorship included oncology (OR, 0.97; 95% CI, 0.81-1.16), ophthalmology (OR, 0.87; 95% CI, 0.72-1.05), and radiation oncology (OR, 0.94 95% CI, 0.56-1.56).

**Conclusions and Relevance:**

The underrepresentation of women among first and last authors in publications of case reports underscores the pervasiveness of sex disparities in medicine. Collaboration and female mentors may be critical instruments in upsetting longstanding practices associated with sex bias. Not all clinical specialties were associated with lower-than-expected female authorship, and further exploration of specialty-specific norms in publication and mentorship may elucidate specific barriers to female authorship.

## Key Points

**Question:**

How are female authors represented in the production of nonresearch medical information across clinical specialties, and what factors may determine their presence?

**Findings:**

In this cross-sectional study of 20 427 case reports, female first (36%) and last (25%) authors were underrepresented in nonresearch publications, and female first authors were associated with female last authors and academic environments. While female author underrepresentation was largely associated with the sex composition of clinical specialties, several predominantly male specialties, including oncology, ophthalmology, optometry, and radiation oncology, were not associated with any specific author sex.

**Meaning:**

Disparities between male and female authors are pervasive even in nonresearch medical publications independent of information content, geography, and specialty.

## Teaser

This cross-sectional study examines the prevalence of female authors of case studies published in medical journals in the United States.

## Introduction

Underrepresentation of women remains prevalent in science and medicine, tainting research practices, career advancement, leadership opportunities, financial compensation, and scientific recognition.^1,2,3,4,5,6,7,8,9,10^ Given the importance of publications in personal and professional development, it is concerning that female authors are underrepresented in biological and medical research publication bylines.^6,8,11,12,13,14^ However, prior evidence supporting this finding has been based on the piecemeal examination of a few, often high-impact, journals of primary research without accounting for academic affiliation, geography, research content, and specialty-specific publishing patterns. Given the focus of past studies on primary research articles, it remains unclear whether sex disparities stem from the conduct of research or authorship practices. Attributing female underrepresentation in primary research articles to authorship practices is difficult because disparities exist in multiple facets of research career development, including greater school debt, higher burnout rates, underrepresentation of women in higher-prestige graduate programs and laboratories, less funding for female investigators from their own institutions and the National Institutes of Health, and faculty promotion.^15,16,17,18,19,20,21,22,23,24^ In addition, while female authors are reportedly scarce in many clinical specialties, it is unclear how author demographic characteristics differ across specialties and whether authorship is associated with the sex composition of specialties.^25,26,27,28,29^ Whether past findings of female underrepresentation are truly pertinent or generalizable to broad authorship practices remains to be clarified.

Case reports are ubiquitous instruments in disseminating knowledge with low barriers to authorship and publication. Given their uniform publication criteria and reporting format, readily achievable authorship contributions, and limited scope, case reports represent a homogeneous subset of the medical literature. Specifically, writing of case reports does not entail extensive training, specialized expertise, reagents, equipment, facilities, and other resources that are generally necessary for performing primary research and are sources of sex bias. We propose that elements unique to case reports make them an ideal venue for discerning sex-specific authorship practices because of a more level opportunity for authorship and the feasibility of adjusting for case report content. Notably, case reports represent a distinct form of clinical information and serve as an educational resource and a source of hypothesis-generating observations. However, case reports remain an uncharted portion of the biomedical corpus, as their production and authorship have not been examined to date. It is unknown whether sex bias exists in such instances of nonresearch medical publications in contrast to reviews, editorials, perspectives, and comments, which are often unsolicited or editorially commissioned and may not undergo peer review, resulting in significant bias.^30^ In this article, we characterize the production of case reports and the sex composition of their authors.

## Methods

All publications indexed in PubMed between 2014 and 2015 classified as a case report under “Article Types” and with at least 1 US author were manually inspected between July 2015 and July 2018. Case reports were extracted by searching for “USA” and state names and abbreviations in author affiliations. Entries that did not provide either the age or sex of the patient being described were excluded, which accounted for approximately 0.5% of all cases reviewed. Studies of more than 3 patients were omitted, as they could be classified as cohort studies and, at some institutions, require institutional review board approval. Our final data set was composed of 20 427 case reports published across 2538 journals. For each case report, journal title, author numbers, patient sex, patient age, patient race, patient ethnicity, and intended purpose were curated after 2 of us analyzed the full texts and a third adjudicated any discrepancies. State of origin was determined from the address of first author affiliations. An academic status was determined by whether the first author affiliation was a teaching hospital or medical school. First and last author sex and author specialty were determined through internet searches and gleaned through news releases, institutional websites, or publicly accessible personal or social network profiles. For 0.4% of cases we used the web application genderapi.io to discern author sex. Author specialty was also determined through bylines if it could not be determined through our initial internet search strategy. The study was exempt from institutional review board review under federal regulation 45 CFR §46.104 because the data were collected from existing records that are publicly available. This study conforms to the Strengthening the Reporting of Observational Studies in Epidemiology (STROBE) reporting guideline for cross-sectional studies.

Author specialty categories were based on specialties and subspecialties defined by the American Board of Medical Specialties as well as nonphysician professional degrees. Specialty categories included allergy and immunology, complementary and alternative medicine (including naturopathy and homeopathy), anesthesia, audiology, cardiology, chiropractic, dentistry, dermatology, endocrinology, family medicine, general surgery, medical genetics, gastroenterology, hematology, infectious disease, neonatology, nephrology, neurology, neurosurgery, nursing, nutrition, obstetrics and gynecology, oncology, ophthalmology, optometry, orthopedic surgery, otolaryngology, palliative medicine, pathology, pharmacy, physician assist, plastic surgery, physical medicine and rehabilitation, podiatry, psychiatry, psychology, physical and occupational therapy, pulmonology, radiology, radiation oncology, rheumatology, social work, speech therapy, sports medicine, urology, and vascular surgery.

We obtained US and state population data through the US Census Bureau website and physician numbers and demographics through the Association of American Medical Colleges Workforce Data portal. Demographic characteristics of nonphysician specialties were obtained from the US Department of Health and Human Services Bureau of Health Workforce and National Center for Health Workforce Analysis.

### Statistical Analysis

The Pearson correlation coefficient and coefficient of determination were calculated to assess the linear correlation and explained variance, respectively, between case report publications and state population, physician numbers, or trainee numbers. Based on the close association between trainee numbers and the production of case reports and the convention of first authors often being junior, we correlated the proportion of female first authors with female trainees across all states to assess the role of geography.^31^ As last authors are typically senior, the proportion of female last authors per state was correlated with active female physician numbers to assess authorship patterns across states. Statistical testing of differences between proportions was performed using the N − 1 χ^2^ test.^32^ β values used to determine odds ratios (ORs) of bibliometric variables and 95% confidence intervals were analyzed by multivariable logistic regression using SPSS statistical software version 23 (IBM). In the multivariable analysis, first author sex was used as the independent variable and state location, academic status, author number, last author sex, reporting purpose, patient age, patient sex, patient race, sex of last author, and author specialty were predictor variables. A 2-sided *P* value of less than .05 was considered statistically significant.

## Results

**Figure 1.**
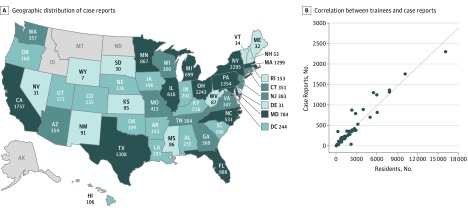
Geographic Distribution of Case Reports A, Heat map portrayal of case report production with dark blue denoting states that produced the most case reports and light blue denoting states that produced the fewest. The number of case reports produced by each state is shown. States that produced fewer than 5 case reports over 2 years were excluded and denoted in gray. B, Correlation between number of residents and case reports per state (*r* = 0.96; *P* < .001).

We analyzed 20 427 case reports published across 2538 journals in 2014 and 2015. The geographic distribution of case reports revealed that anecdotal knowledge production is enriched in select states of variable population size (Figure 1A). We hypothesized that the high concentration of medical schools and postgraduate clinical training programs in particular states could contribute to this phenomenon. Indeed, at the state level, there was a significant correlation between medical trainees and case reports, with 92% of differences in case report numbers explained by number of trainees (*r* = 0.96; *P* < .001) (Figure 1B). State population and physician numbers only explained 64% and 77% of the variance in case reports, and only trainee numbers were independently associated with case reports (physician numbers were excluded due to multicollinearity) based on a multivariable linear regression model (β = 0.15; *P* < .001). Consistent with this, 82% of case reports were produced at academic institutions.

**Figure 2.**
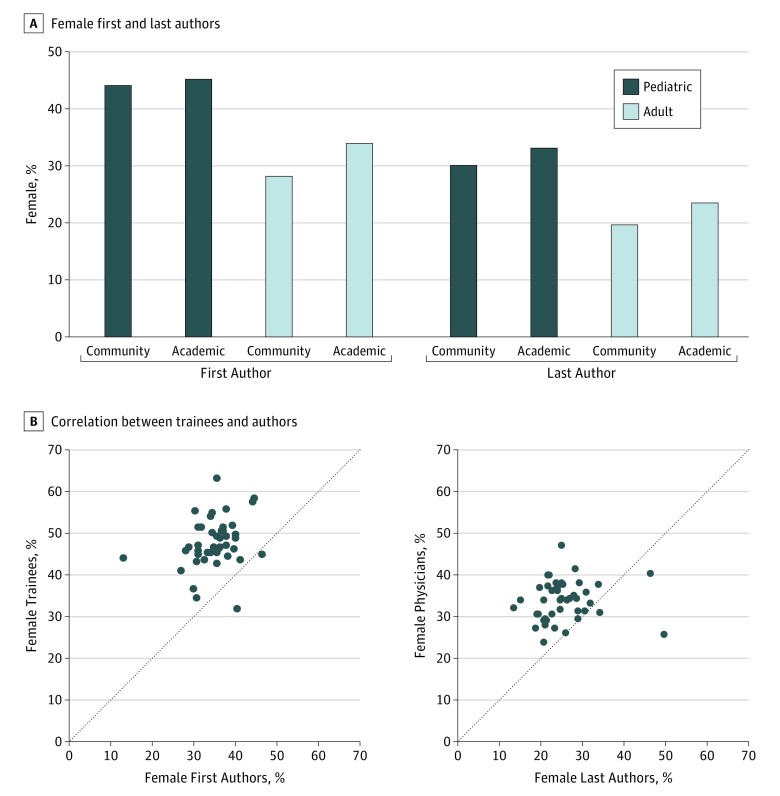
Female Authorship in Case Reports A, Female first and last authors were more prevalent in pediatric cases and in academic settings. B, Correlation plots between the proportion of female trainees or physicians and female first or last authors by state.

In all, 36% and 25% of case reports had a female first and last author, respectively. In comparison, 44% and 34% of US trainees and physicians, respectively, were female according to the 2016 American Medical Association Physician Report. Female first authors in adult case reports were more prevalent in academic environments compared with community settings (34.0% vs 28.2%; difference, 5.8%; 95% CI, 3.8%-7.7%; *P* < .001) (Figure 2A). The difference in incidence between academic and community female first authors in pediatric case reports was not significant (45.2% vs 44.2%; difference, 1.1%; 95% CI, −3.3% to 5.3%; *P* = .63). There was also an increased frequency of female last authors in academic adult (23.4% vs 19.7%; difference, 3.7%; 95% CI, 1.7%-5.4%; *P* < .001) and pediatric (33.1% vs 30.2%; difference, 2.9%; 95% CI, −1.5% to 6.9%; *P* = .19) case reports. The proportions of female first and last authors were universally less than the proportions of female trainees and active female physicians, respectively, across states (Figure 2B).

Larger author teams (OR, 1.02; 95% CI, 1.00-1.03), an academic affiliation (OR, 1.16; 95% CI, 1.06-1.27), and a female last author (OR, 1.58; 95% CI, 1.47-1.70) were significantly associated with female first authorship (Table 1). Compared with the reporting of novel therapies, female first authors were more likely to report technological achievements (OR, 2.15; 95% CI, 1.49-3.11), novel presentations (OR, 1.24; 95% CI, 1.13-1.36), and genetic studies (OR, 1.26; 95% CI, 1.01-1.56). Among patient demographic characteristics, female patients (OR, 1.13; 95% CI, 1.06-1.20) and younger patients (OR, 0.99; 95% CI, 0.99-0.99) were associated with female first authors. Relative to general internal medicine, otorhinolaryngology (OR, 0.77; 95% CI, 0.62-0.95), general surgery (OR, 0.75; 95% CI, 0.60-0.93), gastroenterology (OR, 0.69; 95% CI, 0.56-0.84), and other specialties dominated by male clinicians were less frequently associated with female first authors (Table 2). Notable exceptions included oncology (OR, 0.97; 95% CI, 0.81-1.16), ophthalmology (OR, 0.87; 95% CI, 0.72-1.05), optometry (OR, 1.10; 95% CI, 0.60-2.02), and radiation oncology (OR, 0.94 95% CI, 0.56-1.56), which were not associated with any author sex.

**Table 1.** Multivariable Analysis of Bibliometric Factors Associated With Female First Authors in Case Reports

Author and Patient Characteristics	Female First Author, No. (%)	Adjusted Odds Ratio of Female First Author (95% CI)^a^
Author affiliation		
Community	939 (30.7)	1 [Reference]
Academic	6313 (36.3)	1.16 (1.06-1.27)
No. of authors		1.02 (1.01-1.03)
Sex of last author		
Male	4555 (31.5)	1 [Reference]
Female	2406 (49.9)	1.58 (1.47-1.70)
Reporting purpose		
Novel therapy	878 (28.3)	1 [Reference]
Technology	57 (41.0)	2.15 (1.49-3.11)
Iatrogenic event	435 (26.5)	1.01 (0.88-1.17)
Presentation	5122 (37.7)	1.24 (1.13-1.36)
Genetic	272 (47.7)	1.26 (1.01-1.56)
Adverse drug effect	480 (37.0)	0.98 (0.85-1.14)
Patient age		0.99 (0.99-0.99)
Patient sex		
Male	3522 (33.5)	1 [Reference]
Female	3673 (38.2)	1.13 (1.06-1.20)
Patient race/ethnicity		
White	1005 (40.2)	1 [Reference]
Unknown	5746 (34.6)	1.02 (0.93-1.12)
Asian	49 (36.0)	0.83 (0.57-1.21)
Black	438 (40.9)	1.12 (0.96-1.30)
Hispanic	207 (42.8)	1.13 (0.92-1.39)
American Indian/Alaska Native	11 (45.8)	0.84 (0.37-1.93)

^a^ Multivariable analysis of female first authorship adjusting for state location, academic status, author number, last author sex, reporting purpose, patient age, patient sex, patient race, sex of last author, and author specialty.

**Table 2.** Multivariable Analysis of Female First Authors in Case Reports by Medical Specialty

Medical Specialty	Female First Author, No. (%)	Adjusted Odds Ratio of Female First Author (95% CI)^a^
General medicine	372 (40.5)	1 [Reference]
Allergy and immunology	95 (64.2)	2.64 (1.83-3.80)
Alternative medicine	14 (30.4)	0.80 (0.41-1.53)
Anesthesia	145 (27.0)	0.59 (0.46-0.75)
Audiology	2 (50.0)	1.41 (0.19-10.30)
Cardiology	289 (17.9)	0.35 (0.29-0.42)
Cardiothoracic surgery	86 (18.3)	0.38 (0.29-0.50)
Dermatology	661 (57.7)	1.86 (1.55-2.22)
Dentistry	94 (24.7)	0.51 (0.39-0.66)
Endocrinology	133 (56.1)	1.80 (1.35-2.42)
Emergency medicine	307 (32.5)	0.72 (0.59-0.87)
Otorhinolaryngology	211 (33.1)	0.77 (0.62-0.95)
Family medicine	63 (38.2)	0.96 (0.68-1.36)
Gastroenterology	258 (30.4)	0.69 (0.56-0.84)
Genetics	198 (53.2)	1.37 (1.05-1.79)
Surgery	198 (32.7)	0.75 (0.60-0.93)
Infectious disease	225 (45.3)	1.17 (0.93-1.46)
Nephrology	108 (29.4)	0.63 (0.49-0.83)
Neurology	444 (34.2)	0.75 (0.63-0.89)
Neonatology	28 (41.2)	0.99 (0.59-1.64)
Neurosurgery	86 (11.0)	0.20 (0.16-0.27)
Nursing	197 (82.8)	8.44 (5.81-12.25)
Nutrition	2 (50.0)	1.91 (0.26-14.03)
Obstetrics and gynecology	267 (66.3)	2.62 (2.04-3.37)
Oncology	445 (40.6)	0.97 (0.81-1.16)
Ophthalmology	372 (37.9)	0.87 (0.72-1.05)
Optometry	20 (40.8)	1.10 (0.60-2.02)
Orthopedic surgery	50 (9.2)	0.17 (0.12-0.23)
Pathology	613 (50.4)	1.37 (1.15-1.64)
Palliative medicine	21 (44.7)	1.15 (0.63-2.09)
Pediatric	106 (45.1)	1.41 (1.26-1.58)
Pharmacy	122 (56.0)	1.96 (1.44-2.67)
Plastic surgery	56 (23.8)	0.53 (0.38-0.74)
Physical medicine and rehabilitation	41 (30.6)	0.65 (0.44-0.97)
Podiatry	19 (23.8)	0.48 (0.28-0.82)
Psychiatry	153 (45.7)	1.32 (1.02-1.70)
Physical and occupational therapy	68 (51.1)	1.66 (1.14-2.41)
Pulmonology	150 (30.2)	0.66 (0.53-0.84)
Psychology	59 (49.2)	1.38 (0.94-2.04)
Physician assistant	34 (55.7)	2.46 (1.43-4.22)
Radiology	273 (27.3)	0.55 (0.45-0.67)
Rheumatology	110 (49.5)	1.37 (1.01-1.84)
Radiation oncology	27 (40.3)	0.94 (0.56-1.56)
Sports medicine	10 (23.3)	0.44 (0.21-0.91)
Speech	20 (95.2)	30.29 (4.01-228.61)
Urology	61 (25.4)	0.54 (0.39-0.75)
Vascular surgery	34 (15.2)	0.29 (0.20-0.43)

^a^ Multivariable analysis of female first authorship from Table 1 depicting associations with different medical specialties.

## Discussion

Case reporting is a long-standing clinical tradition abundant in the medical literature, but to date it remains a poorly characterized aspect of knowledge production. In this study, we assessed the production of case reports and demonstrated the underrepresentation of female authorship and its associated factors. This finding was omnipresent across the United States, with the proportion of female first authors being less than the proportion of either female trainees or active physicians in any state. While female authors are known to be less prevalent in primary research articles, it was unknown whether this was completely or partly explained by sex disparities in funding, research team inclusion, subject discipline, research environment, publication practices, or reviewer bias.^33,34,35^ Given the relatively small sample sizes and small numbers of journals examined in past studies, it has also been unclear whether female author underrepresentation was generalizable to a wider landscape of medical journals, particularly in less-read or -cited publishing venues.^8,11,12,13,36^

Case reports are inherently self-evident and require minimal material or infrastructure input. Thus, our systematic approach of studying all case reports published over 2 years in the United States provides an outlook on publishing practices that should be less susceptible to known causes of sex disparities in research and medicine. Case reports represent a widely accessible opportunity for any clinician, including trainees, to contribute to medical knowledge and gain experience in hypothesis generation, project design, scientific writing, and publishing. There may also be tangible benefits to publishing case reports in career advancement, albeit less so than primary research publications.^37^ Thus, our findings that women are underrepresented among first and last authors in such nonresearch publications of clinical information underscores the pervasiveness of sex disparities in medicine and their potential impact.

It is well established that women in academics persistently face barriers owing to disparities in recruitment, authorship, promotion, and pay despite their greater participation in service activities and empirical evidence for improved team performance with greater sex diversity.^34,38,39,40,41,42,43^ Our finding that female first authorship in case reports is associated with academic environments and female last authorship is intriguing, as it may reflect mentorship quality or bias against mentees in different settings. Many complex issues surround the appropriate remedy for sex disparities in science, but increasing diversity among senior teaching or supervisory positions within academic institutions may lead to greater representation of women among first authors and the impartial growth of medical knowledge. This is supported by empirical evidence that women intimately linked to a predominantly female clique are more likely to acquire leadership positions in various organizations.^44^

Female first authors were also associated with team size, which may be due to a higher propensity for forming collaborations.^45,46^ This finding could also indicate that women working in a team are more likely to publish. Larger scientific teams are associated with higher-impact works, and establishing a system of embedding female trainees or junior faculty within larger research networks may engender increased productivity and impact.^47,48^

The association of individual specialties with female first authors often corresponded with sex composition, but it is encouraging that select male-dominated disciplines exhibited no evidence of bias against female authorship. In particular, oncology, ophthalmology, and radiation oncology specialties have a greater proportion of male trainees and active physicians compared with internal medicine, despite exhibiting a relatively equivalent tendency for male and female first authorship. Consistent with this, these specialties have a larger proportion of female authors compared with the proportion of women in the workforce. Unlike the remainder of other specialties, cardiothoracic surgery, emergency medicine, orthopedic surgery, and palliative medicine were associated with a lower OR of female first authors but had a higher proportion of female first and last authors relative to the workforce. This finding suggests that the association of these specialties with male authorship is well explained by the low prevalence of female clinicians rather than bias against female authorship or author byline placement. An in-depth analysis of these specialties may identify cultures or strategies that can be refined for the majority of other specialties where disparities are apparent.

Because the presence of a female first author was associated with reporting on female patients even after controlling for clinician specialty, it is plausible that patient demographic characteristics in case reports are also skewed. There may be practical implications of this, as case reports typically describe novel associations, rare adverse events, and exceptional treatment responses, which may guide the management of conditions. Because case report findings are not widely generalizable, adequate portrayal of patient information and sufficient representation of demographic characteristics are necessary to make accurate inferences, particularly as emerging therapies are associated with sex-specific efficacy or toxicity.^49,50^ Although patient demographic characteristics of case reports have not been clearly elucidated, our findings highlight the potential bias in a source of clinical knowledge commonly assumed to be objective.

### Limitations

This study has limitations. We relied on PubMed for the extraction of case reports, and not all case reports published by US authors may have been identified if they were published in journals that were not indexed. However, PubMed searches within MEDLINE and related databases, which are among the largest repositories of clinical and biomedical literature information and journals that must meet quality standards prior to inclusion. Author demographic data were determined from various sources, and we cannot account for error as a result of inaccurate public information. In addition, our cross-sectional analysis only spanned a 2-year interval, which precluded any analyses of temporal trends. Our study does not clarify whether sex disparities have worsened or improved over time, and it would be of interest for future studies to investigate whether publication practices are indeed malleable.

## Conclusions

This study suggests that authorship disparities by sex are pervasive even in nonresearch medical publications independent of information content, geography, and specialty. The associations of female last authors and team size with female first authors highlight potential methods to promote diversity and impartiality in professional development and the production of clinical knowledge in medicine. Further exploration into the mentoring practices in specialties with less sex disparity in authorship may also identify skills and strategies for reducing bias and its impact among clinicians.

## References

[1] Ma Y, Oliveira DFM, Woodruff TK, Uzzi B Women who win prizes get less money and prestige. Nature. 2019;565(7739):-. doi:10.1038/d41586-019-00091-3 30651627

[2] Nittrouer CL, Hebl MR, Ashburn-Nardo L, Trump-Steele RCE, Lane DM, Valian V Gender disparities in colloquium speakers at top universities. Proc Natl Acad Sci U S A. 2018;115(1):104-108. doi:10.1073/pnas.1708414115 29255050 PMC5776791

[3] Holman L, Stuart-Fox D, Hauser CE The gender gap in science: how long until women are equally represented? PLoS Biol. 2018;16(4):e2004956. doi:10.1371/journal.pbio.2004956 29672508 PMC5908072

[4] Hechtman LA, Moore NP, Schulkey CE, NIH funding longevity by gender. Proc Natl Acad Sci U S A. 2018;115(31):7943-7948. doi:10.1073/pnas.1800615115 30012615 PMC6077749

[5] Glauser W Rise of women in medicine not matched by leadership roles. CMAJ. 2018;190(15):E479-E480. doi:10.1503/cmaj.109-5567 29661820 PMC5903894

[6] Sun GH, Moloci NM, Schmidt K, Maceachern MP, Jagsi R Representation of women as authors of collaborative cancer clinical trials. JAMA Intern Med. 2014;174(5):806-808. doi:10.1001/jamainternmed.2014.250 24664468

[7] Mayer AP, Blair JE, Ko MG, Gender distribution of U.S. medical school faculty by academic track type. Acad Med. 2014;89(2):312-317. doi:10.1097/ACM.0000000000000089 24362384

[8] Erren TC, Groß JV, Shaw DM, Selle B Representation of women as authors, reviewers, editors in chief, and editorial board members at 6 general medical journals in 2010 and 2011. JAMA Intern Med. 2014;174(4):633-635. doi:10.1001/jamainternmed.2013.14760 24566922

[9] Jagsi R, Tarbell NJ, Henault LE, Chang Y, Hylek EM The representation of women on the editorial boards of major medical journals: a 35-year perspective. Arch Intern Med. 2008;168(5):544-548. doi:10.1001/archinte.168.5.544 18332302

[10] Wright AL, Schwindt LA, Bassford TL, Gender differences in academic advancement: patterns, causes, and potential solutions in one US College of Medicine. Acad Med. 2003;78(5):500-508. doi:10.1097/00001888-200305000-00015 12742788

[11] Aakhus E, Mitra N, Lautenbach E, Joffe S Gender and byline placement of co-first authors in clinical and basic science journals with high impact factors. JAMA. 2018;319(6):610-611. doi:10.1001/jama.2017.18672 29450515 PMC5838607

[12] Filardo G, da Graca B, Sass DM, Pollock BD, Smith EB, Martinez MA Trends and comparison of female first authorship in high impact medical journals: observational study (1994-2014). BMJ. 2016;352:i847. doi:10.1136/bmj.i847 26935100 PMC4775869

[13] Jagsi R, Guancial EA, Worobey CC, The “gender gap” in authorship of academic medical literature—a 35-year perspective. N Engl J Med. 2006;355(3):281-287. doi:10.1056/NEJMsa053910 16855268

[14] van Dijk D, Manor O, Carey LB Publication metrics and success on the academic job market. Curr Biol. 2014;24(11):R516-R517. doi:10.1016/j.cub.2014.04.039 24892909

[15] Miller K Deeper in Debt Women and Student Loans. Washington, DC: American Association of University Women; 2017.

[16] Weeden K, Thébaud S, Gelbgiser D Degrees of difference: gender segregation of U.S. doctorates by field and program prestige. Sociol Sci. 2017;4:123-150. doi:10.15195/v4.a6

[17] Langin K When you’re the only woman: the challenges for female Ph.D. students in male-dominated cohorts. Science. October 24, 2018. doi:10.1126/science.caredit.aav8395

[18] Sheltzer JM, Smith JC Elite male faculty in the life sciences employ fewer women. Proc Natl Acad Sci U S A. 2014;111(28):10107-10112. doi:10.1073/pnas.1403334111 24982167 PMC4104900

[19] Sege R, Nykiel-Bub L, Selk S Sex differences in institutional support for junior biomedical researchers. JAMA. 2015;314(11):1175-1177. doi:10.1001/jama.2015.8517 26372589

[20] Jena AB, Khullar D, Ho O, Olenski AR, Blumenthal DM Sex differences in academic rank in US medical schools in 2014. JAMA. 2015;314(11):1149-1158. doi:10.1001/jama.2015.10680 26372584 PMC4665995

[21] Oliveira DFM, Ma Y, Woodruff TK, Uzzi B Comparison of National Institutes of Health grant amounts to first-time male and female principal investigators. JAMA. 2019;321(9):898-900. doi:10.1001/jama.2018.21944 30835300 PMC6439593

[22] Carr PL, Raj A, Kaplan SE, Terrin N, Breeze JL, Freund KM Gender differences in academic medicine: retention, rank, and leadership comparisons from the National Faculty Survey. Acad Med. 2018;93(11):1694-1699. doi:10.1097/ACM.0000000000002146 29384751 PMC6066448

[23] Dwyer RE, Hodson R, McLoud L Gender, debt, and dropping out of college. Gend Soc. 2013;27(1):30-55. doi:10.1177/0891243212464906 23626403 PMC3633219

[24] Evans TM, Bira L, Gastelum JB, Weiss LT, Vanderford NL Evidence for a mental health crisis in graduate education. Nat Biotechnol. 2018;36(3):282-284. doi:10.1038/nbt.4089 29509732

[25] Long MT, Leszczynski A, Thompson KD, Wasan SK, Calderwood AH Female authorship in major academic gastroenterology journals: a look over 20 years. Gastrointest Endosc. 2015;81(6):1440-1447.e3. doi:10.1016/j.gie.2015.01.032 25887727

[26] Yun EJ, Yoon DY, Kim B, Closing the gender gap: increased female authorship in *AJR* and *Radiology*. AJR Am J Roentgenol. 2015;205(2):237-241. doi:10.2214/AJR.14.14225 26204270

[27] Miller J, Chuba E, Deiner S, DeMaria S Jr, Katz D Trends in authorship in anesthesiology journals. Anesth Analg. 2018. doi:10.1213/ANE.0000000000003949 30418237

[28] Fishman M, Williams WA II, Goodman DM, Ross LF Gender differences in the authorship of original research in pediatric journals, 2001-2016. J Pediatr. 2017;191:244-249.e1. doi:10.1016/j.jpeds.2017.08.044 29033241

[29] Zhang S, Kim HY, Hill RES, Veledar E, Chen SC A ten-year comparison of women authorship in U.S. dermatology literature, 1999 vs. 2009. Int J Womens Dermatol. 2017;3(1)(suppl):S58-S61. doi:10.1016/j.ijwd.2017.02.012 28492041 PMC5419057

[30] Silver JK, Poorman JA, Reilly JM, Spector ND, Goldstein R, Zafonte RD Assessment of women physicians among authors of perspective-type articles published in high-impact pediatric journals. JAMA Netw Open. 2018;1(3):e180802. doi:10.1001/jamanetworkopen.2018.0802 30646033 PMC6324294

[31] Fontanarosa P, Bauchner H, Flanagin A Authorship and team science. JAMA. 2017;318(24):2433-2437. doi:10.1001/jama.2017.19341 29279909

[32] Campbell I Chi-squared and Fisher-Irwin tests of two-by-two tables with small sample recommendations. Stat Med. 2007;26(19):3661-3675. doi:10.1002/sim.2832 17315184

[33] van der Lee R, Ellemers N Gender contributes to personal research funding success in the Netherlands. Proc Natl Acad Sci U S A. 2015;112(40):12349-12353. doi:10.1073/pnas.1510159112 26392544 PMC4603485

[34] Moss-Racusin CA, Dovidio JF, Brescoll VL, Graham MJ, Handelsman J Science faculty’s subtle gender biases favor male students. Proc Natl Acad Sci U S A. 2012;109(41):16474-16479. doi:10.1073/pnas.1211286109 22988126 PMC3478626

[35] Helmer M, Schottdorf M, Neef A, Battaglia D Gender bias in scholarly peer review. Elife. 2017;6:6. doi:10.7554/eLife.21718 28322725 PMC5360442

[36] González-Alvarez J Author gender in *The Lancet* journals. Lancet. 2018;391(10140):2601. doi:10.1016/S0140-6736(18)31139-5 30070215

[37] Bavdekar SB, Tullu MS Research publications for academic career advancement: an idea whose time has come. but is this the right way? J Postgrad Med. 2016;62(1):1-3. doi:10.4103/0022-3859.39180 26732190 PMC4944322

[38] Buffington C, Harris BC, Jones C, Weinberg BA STEM training and early career outcomes of female and male graduate students: evidence from UMETRICS data linked to the 2010 Census. Am Econ Rev. 2016;106(5):333-338. doi:10.1257/aer.p20161124 27231399 PMC4876811

[39] Gumpertz M, Durodoye R, Griffith E, Wilson A Retention and promotion of women and underrepresented minority faculty in science and engineering at four large land grant institutions. PLoS One. 2017;12(11):e0187285. doi:10.1371/journal.pone.0187285 29091958 PMC5665535

[40] Shen YA, Shoda Y, Fine I Too few women authors on research papers in leading journals. Nature. 2018;555(7695):165-165. doi:10.1038/d41586-018-02833-1 32095010

[41] John AM, Gupta AB, John ES, Lopez SA, Lambert WC A gender-based comparison of promotion and research productivity in academic dermatology. Dermatol Online J. 2016;22(4):13030/qt1hx610pf. 27617455

[42] Nielsen MW, Alegria S, Börjeson L, Opinion: gender diversity leads to better science. Proc Natl Acad Sci U S A. 2017;114(8):1740-1742. doi:10.1073/pnas.1700616114 28228604 PMC5338420

[43] Guarino CM, Borden VMH Faculty service loads and gender: are women taking care of the academic family? Res High Educ. 2017;58(6):672-694. doi:10.1007/s11162-017-9454-2

[44] Yang Y, Chawla NV, Uzzi B A network’s gender composition and communication pattern predict women’s leadership success. Proc Natl Acad Sci U S A. 2019;116(6):2033-2038. doi:10.1073/pnas.1721438116 30670641 PMC6369753

[45] Iglič H, Doreian P, Kronegger L, Ferligoj A With whom do researchers collaborate and why? Scientometrics. 2017;112(1):153-174. doi:10.1007/s11192-017-2386-y 28725095 PMC5486904

[46] Araújo EB, Araújo NAM, Moreira AA, Herrmann HJ, Andrade JS Jr Gender differences in scientific collaborations: women are more egalitarian than men. PLoS One. 2017;12(5):e0176791. doi:10.1371/journal.pone.0176791 28489872 PMC5425184

[47] Wuchty S, Jones BF, Uzzi B The increasing dominance of teams in production of knowledge. Science. 2007;316(5827):1036-1039. doi:10.1126/science.1136099 17431139

[48] Hsiehchen D, Espinoza M, Hsieh A Multinational teams and diseconomies of scale in collaborative research. Sci Adv. 2015;1(8):e1500211. doi:10.1126/sciadv.1500211 26601251 PMC4643764

[49] Özdemir BC, Csajka C, Dotto GP, Wagner AD Sex differences in efficacy and toxicity of systemic treatments: an undervalued issue in the era of precision oncology. J Clin Oncol. 2018;36(26):2680-2683. doi:10.1200/JCO.2018.78.3290 30004815

[50] Conforti F, Pala L, Bagnardi V, Cancer immunotherapy efficacy and patients’ sex: a systematic review and meta-analysis. Lancet Oncol. 2018;19(6):737-746. doi:10.1016/S1470-2045(18)30261-4 29778737

